# Rifampicin-soaked versus bovine prostheses in the management of aortic graft infections: a retrospective single-center cohort study

**DOI:** 10.1007/s00423-026-04349-z

**Published:** 2026-10-23

**Authors:** Viktoria Weronika Strzoda, Miroslav Dimitrov Yordanov, Alexander Oberhuber, Abdulhakim Ibrahim

**Affiliations:** Department of Vascular and Endovascular Surgery, University Hospital Münster, Albert-Schweitzer-Campus 1, 48149 Münster, Germany

**Keywords:** Aortic graft infection, rifampicin-soaked graft, bovine graft, Candida, prosthetic graft infection

## Abstract

**Background:**

Aortic vascular graft and endograft infections (aortic VGEI) remain a rare but life-threatening complication following aortic reconstruction. This retrospective study compares outcomes between bovine pericardial graft prostheses and rifampicin-soaked Dacron grafts.

**Methods:**

In this retrospective single-center cohort study, we analyzed 62 patients between January 2018 and December 2025 for aortic vascular graft or endograft infection requiring graft explantation and in-situ reconstruction. Patients received either a bovine pericardial graft (n=21) or a rifampicin-soaked Dacron prosthesis (n=41). Survival, treatment failure, perioperative variables, and microbiology were analyzed.

**Results:**

Patients in the bovine and rifampicin groups were comparable in terms of age (72.57 vs. 73.58 years, p = 0.685) and sex distribution (85.71% vs. 73.17% male, p = 0.346). BMI, preoperative dialysis, and aortic clamping time were higher in the bovine group. Four treatment failures, defined as persistent or recurrent infection of the reconstructed aortic segment, occurred during follow-up; all were observed after reconstruction with rifampicin-soaked Dacron (4/41, 9.8%), whereas no treatment failure occurred in the bovine pericardial group (0/21). In-hospital mortality was comparable between groups (n=3:14.3% vs. n=8: 19.5%, p = 0.735). Kaplan–Meier analysis demonstrated no significant difference in survival (p = 0.256) or treatment failure rate (p = 0.833). Candida spp. were the most frequent pathogens (27.8%) and significantly associated with secondary aorto-enteric fistula (p=0.003).

**Conclusion:**

Survival and treatment failure rates were comparable, with a trend toward fewer infection-related complications after bovine pericardial grafting. Despite use in more complex cases, bovine grafts may represent a promising biological alternative.

## Introduction

Aortic vascular graft and endograft infection (aortic VGEI) is a rare but life-threatening complication following both open and endovascular aortic repair, with reported incidences of 0.6–3% after open procedures and below 1% after endovascular repair [1, 2]. Clinical presentation ranges from non-specific symptoms such as fever and weight loss to severe manifestations including sepsis and secondary aorto-enteric fistula. In contemporary cohorts of aortic vascular graft and endograft infection, in-hospital mortality ranges from approximately 19% to 48%, with septic shock and aorto-enteric fistula consistently identified as adverse prognostic factors. Secondary aorto-enteric fistula itself has been associated with an in-hospital mortality of approximately 27% [3–7]. Staphylococcus aureus and coagulase-negative staphylococci are the most common pathogens, though Gram-negative bacteria and fungi, particularly Candida species, are also frequently isolated [4].

The optimal surgical management of aortic VGEI remains controversial. Graft explantation followed by in-situ reconstruction is considered the preferred definitive treatment in patients suitable for surgery, as conservative treatment alone is generally associated with poorer long-term outcomes [2, 8]. Nevertheless, conservative or graft-preserving management with prolonged targeted antimicrobial therapy may be considered in carefully selected frail patients, those with prohibitive operative risk, or in technically complex cases in which complete graft explantation is not feasible. A variety of graft materials are available, each with specific advantages and limitations. Autologous veins and cryopreserved allografts achieve the lowest treatment failure rates but are often limited by availability or technical feasibility [2]. Rifampicin-soaked prostheses are widely used in emergency settings due to immediate availability and temporary antimicrobial protection, though treatment failures and resistance remain concerns [1]. Biologic grafts such as bovine pericardial grafts have been introduced more recently and show promising results, with reported treatment failure rates between 0 and 6% and perioperative mortality around 15–20% [4, 9, 10]. However, their use in complex aortic segments requiring multiple branch reconstructions (e.g. thoraco-abdominal or arch procedures) may be limited by the need to intraoperatively construct several individual pericardial grafts. This is technically demanding and time-consuming in an already high-risk setting [11]. Moreover, case series using self-made and prefabricated bovine pericardial grafts have documented occasional graft-related mechanical complications, including kinking and anastomotic stenosis. This emphasises the necessity for meticulous sizing and conduit routing [12]. 

The present study aims to compare perioperative outcomes, treatment failure rates, and survival between bovine pericardial grafts and rifampicin-soaked Dacron prostheses in patients undergoing secondary reconstruction for aortic VGEI at a tertiary referral center.

## Materials and methods

### Study design and patient population

This is a retrospective, single-center cohort study. The study was conducted and reported in accordance with the Strengthening the Reporting of Observational Studies in Epidemiology (STROBE) statement. All patients treated for aortic VGEI between January 2018 and December 2025 were screened for eligibility according to the predefined inclusion and exclusion criteria [13]. The cohort included patients with aortic VGEI involving the infrarenal abdominal aorta or aortoiliac segment following previous open aortic reconstruction or endovascular aneurysm repair. Patients treated conservatively, with graft-preserving procedures, extra-anatomical reconstruction, or other conduit materials were not included in this comparative analysis. Patients were identified through the hospital’s coding system. A total of 62 patients underwent explantation of infected grafts and in-situ reconstruction. Reconstruction was carried out with either a bovine pericardial graft (*n* = 21) or a rifampicin-soaked Dacron prosthesis (*n* = 41). Patients had undergone their primary aortic repair (open or endovascular) either at external institutions or at our center prior to referral. Patients were managed by a multidisciplinary team involving vascular surgeons, infectious diseases specialists, radiologists, anesthesiologists, and intensive care physicians. Diagnostic evaluation, operative strategy, conduit selection, and antimicrobial treatment were determined jointly according to the anatomical extent of infection, microbiological findings, haemodynamic stability, and individual operative risk. Institutional Review Board approval was not required for this study. Patients with aortic VGEI complicated by a secondary aorto-enteric fistula were retained in the cohort but analyzed as a distinct clinical subgroup from patients without enteric involvement.

### Operative urgency

Operative urgency was categorized according to the timing of the procedure. Emergency surgery was defined as immediate operative treatment required because of the patient’s acute clinical condition, whereas urgent surgery was defined as surgery performed within 24 h of the decision to operate.

### Potential bias

Because conduit selection was not randomized and evolved during the study period, confounding by indication and treatment era could not be excluded. These potential sources of bias were considered when interpreting between-group comparisons.

### Definition of aortic graft infection and diagnostic routine

**Fig. 1:**
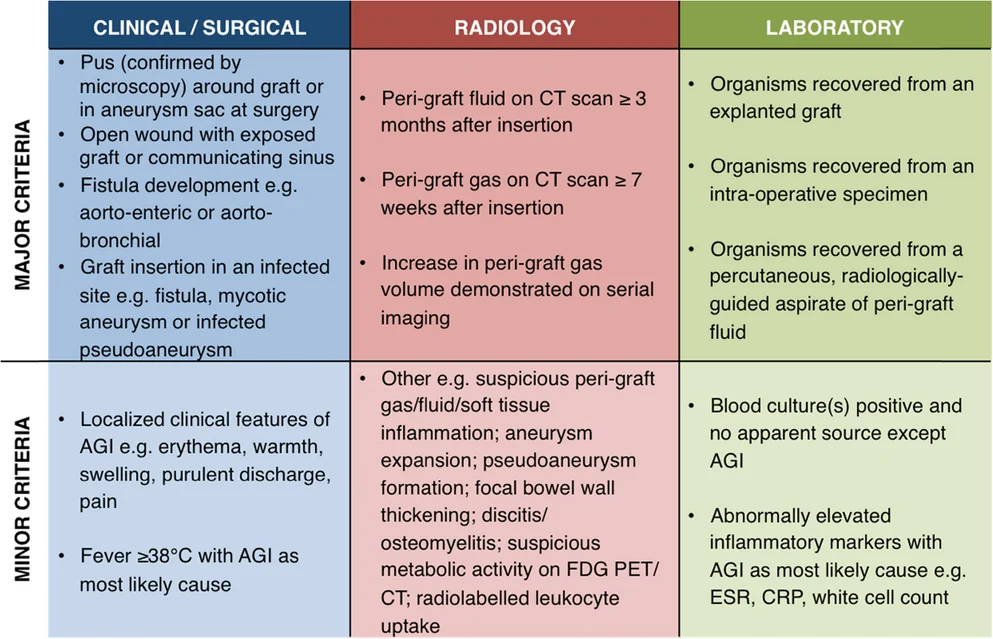
MAGIC criteria for diagnosing aortic vascular graft and endograft infection (aortic VGEI). Diagnosis requires one major criterion plus any additional criterion from another diagnostic category

Aortic vascular graft and endograft infection (aortic VGEI) was defined according to the Management of Aortic Graft Infection Collaboration (MAGIC) criteria, which were developed by a multidisciplinary expert consensus to standardize case definitions and improve diagnostic accuracy. According to these criteria, the suspicion of aortic VGEI is established when one major criterion or two minor criteria from three different diagnostic categories (clinical/surgical, imaging, and laboratory) are fulfilled. A definitive diagnosis requires at least one major criterion combined with an additional criterion, irrespective of category. Major criteria include, for example, a positive microbiological culture from graft or perigraft tissue and characteristic imaging findings such as persistent peri-graft gas or fluid on computed tomography more than seven weeks after implantation (Fig. 1) [1, 14].

PET-CT was performed in all patients as part of the diagnostic work-up. In all included cases, infection involved the central infrarenal aortic graft or endograft segment, with or without extension into the aortoiliac reconstruction; isolated iliac graft infections were excluded. Upper gastrointestinal endoscopy (esophagogastroduodenoscopy, EGD) was routinely performed in cases with suspected secondary aorto-enteric fistula to confirm or exclude direct communication between the aortic graft and the gastrointestinal tract [14]. 

### Inclusion and exclusion criteria

Patients with incomplete clinical records or without confirmed graft infection were excluded. Infections were confirmed based on intraoperative findings, positive microbiological cultures, or fulfillment of MAGIC criteria [14]. Only patients with aortic VGEI fulfilling the MAGIC criteria for a confirmed diagnosis were included. Intraoperative findings, microbiological cultures, laboratory results, and imaging findings were used to determine fulfillment of the MAGIC criteria. Patients with isolated iliac graft infection without involvement of the central aortic segment were excluded. In addition, patients who underwent stent graft explantation due to symptomatic stent graft occlusion, stent graft dislocation, or progressive endoleak were excluded from the study. Patients with prosthetic graft infections after open infrarenal aortic repair (AAA or PAD) or after EVAR for infrarenal abdominal aortic aneurysm were included.

### Data collection

Demographic characteristics, comorbidities, characteristics of the primary aortic procedure, clinical presentation, operative urgency, surgical and perioperative variables, microbiological findings, postoperative complications, antimicrobial treatment, mortality, treatment failure, and follow-up data were retrospectively extracted from the electronic hospital records. Operative reports were additionally reviewed to determine procedural characteristics, intraoperative findings, and the type of reconstruction performed. Microbiological data were obtained from documented preoperative blood cultures and intraoperative swabs and tissue specimens. Follow-up information was obtained from available outpatient documentation, correspondence from referring or primary-care physicians, and subsequent hospital records.

### Endpoints

The primary endpoint was treatment failure following graft explantation and in-situ reconstruction. Treatment failure was defined as persistent or recurrent infection involving the reconstructed aortic segment after the index reconstruction, based on compatible clinical, laboratory, microbiological, and/or imaging findings. In the present cohort, all treatment failures represented recurrent infection after the index reconstruction. Secondary endpoints included in-hospital mortality and major postoperative complications such as the requirement for postoperative renal replacement therapy, graft occlusion, limb amputation, and the need for surgical reintervention. Renal complications were recorded according to documented preoperative dialysis dependency and postoperative dialysis requirement. Preoperative dialysis was considered a baseline comorbidity, whereas postoperative dialysis was recorded as a clinically relevant postoperative renal complication. Unplanned surgical reintervention was defined as any repeat operation required for a postoperative complication, including bleeding, graft thrombosis or occlusion. In-hospital mortality was defined as death from any cause occurring during the index hospitalization, irrespective of the time elapsed since surgery.

### Surgical technique

**Fig. 2:**
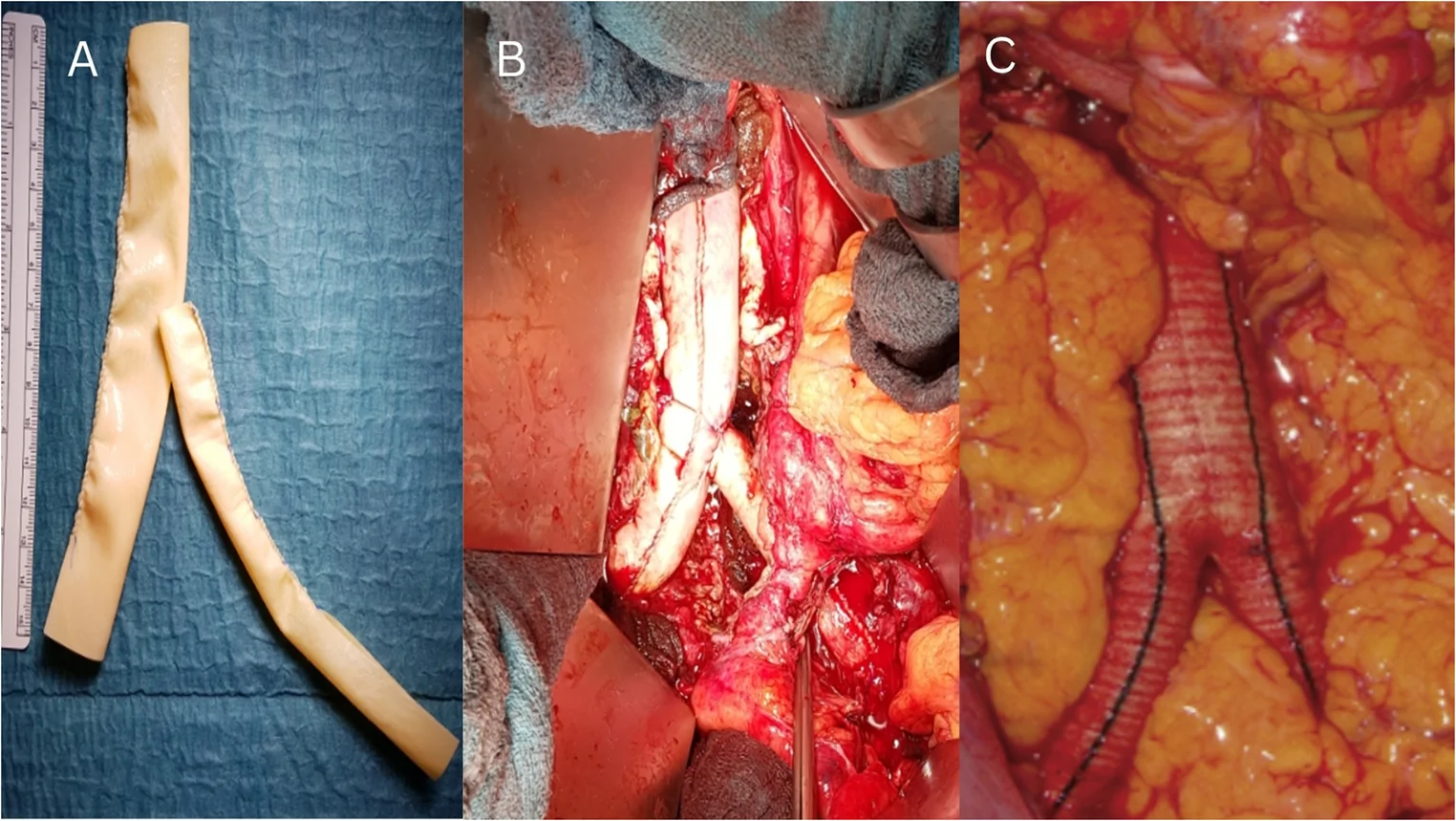
(**A**, **B**) Custom-made prosthesis made of bovine pericardium, (**C**) Implanted rifampicin-soaked prosthesis

All procedures were performed via open surgical access under general anesthesia. After removal of the infected graft and excessive debridement an in-situ reconstruction was performed. Bovine pericardial grafts were hand-sewn intraoperatively from pericardial patches (Fig. 2). Immediately before implantation, Dacron prostheses were soaked for approximately 10 min in a rifampicin solution prepared from 300 mg rifampicin powder dissolved in 50 mL of 0.9% saline, and were then implanted without delay to achieve optimal local antimicrobial effect. Omentoplasty was routinely performed whenever technically feasible to provide additional graft coverage and local infection control. Conduit selection was based on the operating surgeon’s assessment of haemodynamic stability, anatomical complexity, contamination severity, and the time required for graft preparation. Rifampicin-soaked Dacron prostheses were predominantly used during the earlier study period, whereas bovine pericardial grafts were increasingly adopted later as institutional experience with this technique evolved.

### Antimicrobial treatment

Antimicrobial therapy was managed in consultation with the institutional antimicrobial stewardship/infectious diseases team. Preoperative blood cultures were obtained in all patients before initiation of antimicrobial therapy. Intravenous antimicrobial treatment was administered during the inpatient period and subsequently adapted according to microbiological findings and susceptibility testing from blood cultures and intraoperative specimens. Following discharge, prolonged oral antimicrobial therapy was generally continued, with treatment individualized according to the identified pathogen and clinical course. Antifungal therapy was administered when fungal pathogens were identified.

### Follow-up

After hospital discharge, follow-up consisted of clinical assessment, laboratory testing, and duplex ultrasonography. Computed tomography was not performed routinely; CT imaging was obtained when clinical, laboratory, or ultrasonographic findings raised suspicion of persistent or recurrent infection, particularly when new or increasing perigraft fluid collections were detected. Approximately one week after completion of the planned antimicrobial treatment course, an early clinical and laboratory reassessment was recommended, generally through the treating or primary-care physician. Thereafter, follow-up was recommended at approximately three-month intervals initially, followed by six-month intervals and, in clinically stable patients, annual follow-up. Additional cross-sectional imaging or microbiological investigations were performed when recurrent infection was suspected.

### Study size

No formal sample-size calculation was performed because of the retrospective nature of the study and the rarity of aortic vascular graft and endograft infection. The study cohort comprised all patients fulfilling the predefined inclusion criteria during the study period.

### Statistical analysis

Data analysis was performed using IBM SPSS Statistics (IBM Corp., Armonk, NY, USA). Continuous variables are presented as mean ± standard deviation and compared using Student’s t-test or Mann–Whitney U test, as appropriate. Categorical variables were compared using chi-square or Fisher’s exact test. Overall survival and freedom from treatment failure were analyzed using Kaplan–Meier estimates and compared using the log-rank test. Overall survival was calculated from the date of graft explantation and in-situ reconstruction to death from any cause. Freedom from treatment failure was calculated from the date of surgery to the first documented treatment failure; patients without treatment failure were censored at the date of death or last available follow-up. Event coding, follow-up dates, and censoring were verified before the final Kaplan–Meier and log-rank analyses. The four treatment failures occurred after 17–84 months following reconstruction. Mean follow-up was 14.60 ± 27.06 months in the rifampicin-soaked Dacron group and 5.57 ± 11.35 months in the bovine pericardial group. A p-value of < 0.05 was considered statistically significant.

## Results

From January 2018 until December 2025, 62 patients underwent secondary reconstruction for aortic graft infection at our institution. Twenty-one patients (33.9%) were treated with bovine pericardial grafts and 41 patients (66.1%) with rifampicin-soaked Dacron prostheses.

Male sex predominated in both groups. Body mass index (BMI) was significantly higher in the bovine group compared with the rifampicin group (25.84 ± 4.80 vs. 21.31 ± 9.07 kg/m2, *p* = 0.037). Preoperative dialysis dependency was also significantly more frequent in the bovine group (23.8% vs. 4.9%, *p* = 0.039). Other comorbidities such as hypertension, COPD, or peripheral arterial disease (PAD) did not differ significantly (Table 1).

**Table 1:** Baseline characteristics of the study population

Demographic characteristics	Total (*n* = 62)	Bovine (*n* = 21)	Rifampicin (*n* = 41)	*P*-value
Age (years), mean ± SD	72.85 ± 9.2	72.57 ± 8.55	73.58 ± 9.59	0.685
Male sex, n (%)	48 (77.42)	18 (85.71)	30 (73.17)	0.346
BMI kg/m², mean ± SD	22.17 ± 8.2	25.84 ± 4,80	21.31 ± 9,07	**0.037**
Medical history, n (%)				
Arterial hypertension, n (%)	38 (61.29)	9 (42.86)	29 (70.73)	1
Preoperative atrial fibrillation, n (%)	10 (16.13)	4 (19.05)	6 (14.63)	0.716
COPD GOLD III - IV, n (%)	13 (20.97)	3 (14.28)	10 (24,39)	0.514
NYHA III-IV, n (%)	9 (14.52)	2 (9.52)	7 (17.07)	0.479
Smoking, n (%)	26 (41.93)	7 (33.33)	19 (46.34)	0.181
PAD, n (%)	20 (32.26)	6 (28.57)	14 (34.15)	0.561
Preoperative dialysis, n (%)	7 (11.29)	5 (23.81)	2 (4.88)	**0.039**

33 of the patients (53.2%) had previously undergone open aortic repair, whereas the remaining 29 patients (46.8%) had received an endovascular procedure (EVAR) as their index operation (Table 2). Within the bovine pericardial group, 9 patients (42.9%) had a previous open procedure and 12 patients (57.1%) had undergone endovascular repair. In the rifampicin group, 24 patients (58.5%) had a prior open procedure and 17 patients (41.5%) an endovascular repair. Patients who had received prior endovascular repair typically presented with suprarenal fixation of the stent graft by proximal anchoring barbs or hooks, as documented in the operative reports. Secondary reconstruction was performed urgently in 33 of 62 patients (53.2%) and as an emergency procedure in 29 patients (46.8%) (Table 2).

**Table 2:** Primary surgical characteristics and surgical urgency of the study population

Primary surgical characteristics, *n* (%)	Total (*n* = 62)	Bovine (*n* = 21)	Rifampicin (*n* = 41)	*P*-value
Open procedure, n (%)	33 (53.2)	9 (42.9)	24 (58.5)	0.289
Endovascular procedure (EVAR), n (%)	29 (46.8)	12 (57.1)	17 (41.5)	0.289
Surgical urgency, n (%)				
Urgent, n (%)	33 (53.2)	14 (66.7)	19 (46.3)	0.18
Emergency, n (%)	29 (46.8)	7 (33.3)	22 (53.7)	0.18

A secondary aorto-enteric fistula was identified in 27 patients (43.55%), including 10 patients (47.62%) in the bovine group and 17 patients (41.46%) in the rifampicin group, without a significant difference between groups (*p* = 0.789). The mean duration of preoperative antibiotic therapy was 4.2 ± 9.02 days overall and did not differ significantly between the bovine and rifampicin groups (4.52 ± 5.66 vs. 3.85 ± 9.75 days, *p* = 0.773).

Intraoperative aortic clamping time was significantly longer in patients treated with bovine grafts compared with rifampicin-soaked prostheses (79.24 ± 99.55 vs. 39.17 ± 52.07 min, *p* = 0.041). Suprarenal aortic clamping was performed in 29.03% of patients overall (28.57% in the bovine group vs. 29.27% in the rifampicin group, *p* = 1). Supravisceral clamping was required in 45.16% of cases (38.09% vs. 48.78%, *p* = 0.59). The requirement for blood product transfusion, including red blood cell, platelet concentrate, and fresh frozen plasma units, did not differ significantly between the two groups (Table 3).

**Table 3:** Pre-, intra- and postoperative characteristics of the study population

Preoperative surgical characteristics, *n* (%)	Total (*n* = 62)	Bovine (*n* = 21)	Rifampicin (*n* = 41)	*p*-value
Aortoenteric fistula, n (%)	27 (43.55)	10(47.62)	17 (41.46)	0,789
Preoperative antibiotic therapy (days), mean ± SD	4.2 ± 9.02	4.52 ± 5.66	3.85 ± 9.75	0,773
Intraoperative surgical characteristics, n (%)				
Aortic clamping time, mean ± SD	52.5 ± 75.41	79.24 ± 99.55	39.17 ± 52.07	**0.041**
Suprarenal aortic clamping, n (%)	18 (29.03)	6 (28.57)	12 (29.27)	1
Supravisceral aortic clamping, n (%)	28 (45.16)	8 (38.09)	20 (48.78)	0.59
RBC transfusion units, mean ± SD	6.70 ± 8.3	5.33 ± 7.03	6.80 ± 8.30	0.49
Platelet concentrate units, mean ± SD	1.31 ± 6.17	0,42 ± 1.43	1.51 ± 7.02	0.489
Fresh frozen plasma units, mean ± SD	3.9 ± 5.35	4.14 ± 6.99	3.54 ± 3.89	0.668
Bowel surgery, n (%)	16 (25.81)	4 (19.05)	12 (29.27)	0.541
Postoperative characteristics, n (%)				
Wound healing disorder, n (%)	4 (6.45)	2 (9.52)	2 (4.87)	0.599
Amputation, n (%)	1 (1,61)	0 (0)	1 (2.44)	1
Occlusion, n (%)	9 (14.52)	3 (14.28)	6 (14.63)	1
Postoperative dialysis n (%)	9 (14.52)	4 (19.05)	5 (12.19)	0.472
Reinfection after graft explantation and in-situ reconstruction, n (%)	4 (6.45)	0 (0)	4 (9.76)	0.562
Pneumonia, n (%)	1 (1.61)	1 (4.76)	0 (0)	0.339
Wound infection, n (%)	9 (14.52)	2 (9.52)	7 (17.07)	0.705
ICU stay (days), mean ± SD	10.6 ± 14.88	8.09 ± 17.89	10.26 ± 12.04	0.572
30-day in-hospital mortality, n (%)	11 (17.74)	3 (14.28)	8 (19.51)	0.735
Postoperative length of stay (days), mean ± SD	25.51 ± 23.98	29.70 ± 32.54	21.78 ± 16.50	0.21

Among the 27 patients with confirmed secondary aorto-enteric fistula, concomitant bowel management was performed in all cases. Sixteen patients (25.81% of the overall cohort) underwent intestinal resection, whereas the remaining patients were treated with primary suture repair of the affected bowel segment. There was no significant difference between groups (*p* = 0.541). Postoperatively, wound healing disorders occurred in 4 patients (6.45%), graft occlusion in 9 patients (14.52%), postoperative dialysis in 9 patients (14.52%), and limb amputation in 1 patient (1.61%), without significant differences between groups. All patients received prolonged postoperative antimicrobial therapy under infectious diseases guidance. Treatment was targeted to the identified microorganisms and susceptibility profiles as microbiological results were available. Mean ICU stay was 10.6 ± 14.88 days and did not differ significantly between groups (8.09 ± 17.89 vs. 10.26 ± 12.04 days, *p* = 0.572). Mean postoperative hospital stay was 25.51 ± 23.98 days overall and was comparable between groups (29.70 ± 32.54 vs. 21.78 ± 16.50 days, *p* = 0.21).

Tracheostomy was required in three patients (4.8%). Two patients underwent dilatational tracheostomy and one patient underwent percutaneous tracheostomy. All three patients had been reconstructed with a rifampicin-soaked Dacron prosthesis. In-hospital mortality occurred in 11 patients (17.74%) overall, including 3 patients (14.28%) in the bovine group and 8 patients (19.51%) in the rifampicin group, without a significant difference between groups (*p* = 0.735). Most deaths were disease-related, occurring in the early postoperative phase due to multiorgan failure or septic complications following graft infection.

**Fig. 3:**
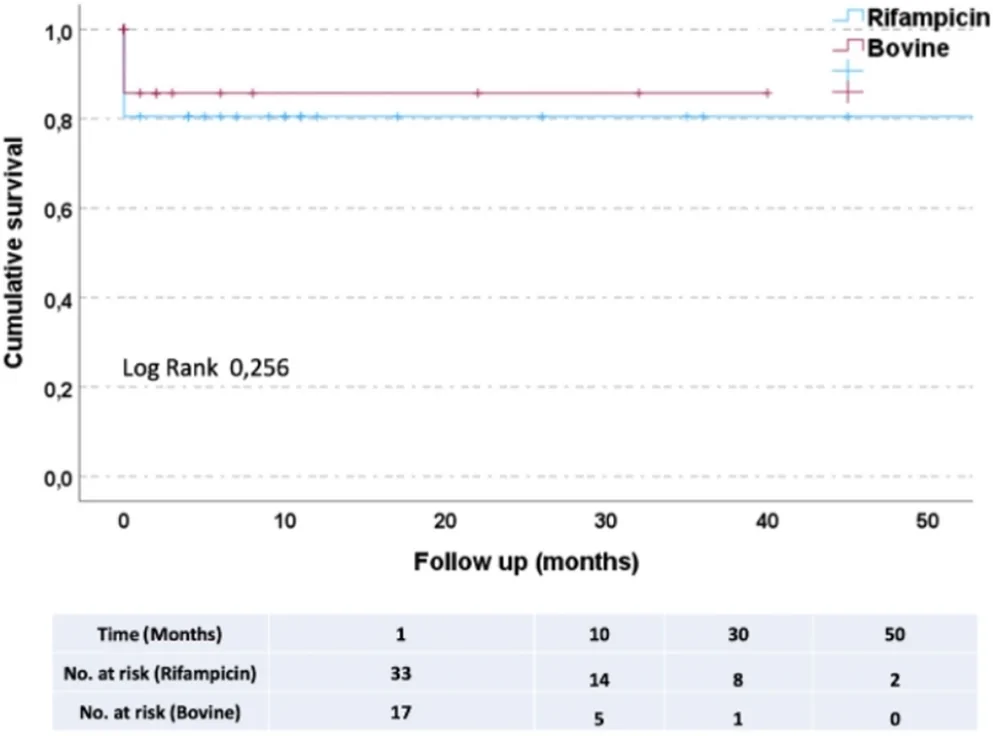
Kaplan-Meier curve of survival rate

The mean follow-up duration was 11.33 ± 24.24 months (rifampicin: 14.60 ± 27.06 vs. bovine: 5.57 ± 11.35 months). Kaplan–Meier analysis demonstrated no significant difference in overall survival between patients treated with bovine pericardial grafts and rifampicin-soaked prostheses (*p* = 0.256) (Fig. 3).

**Fig. 4:**
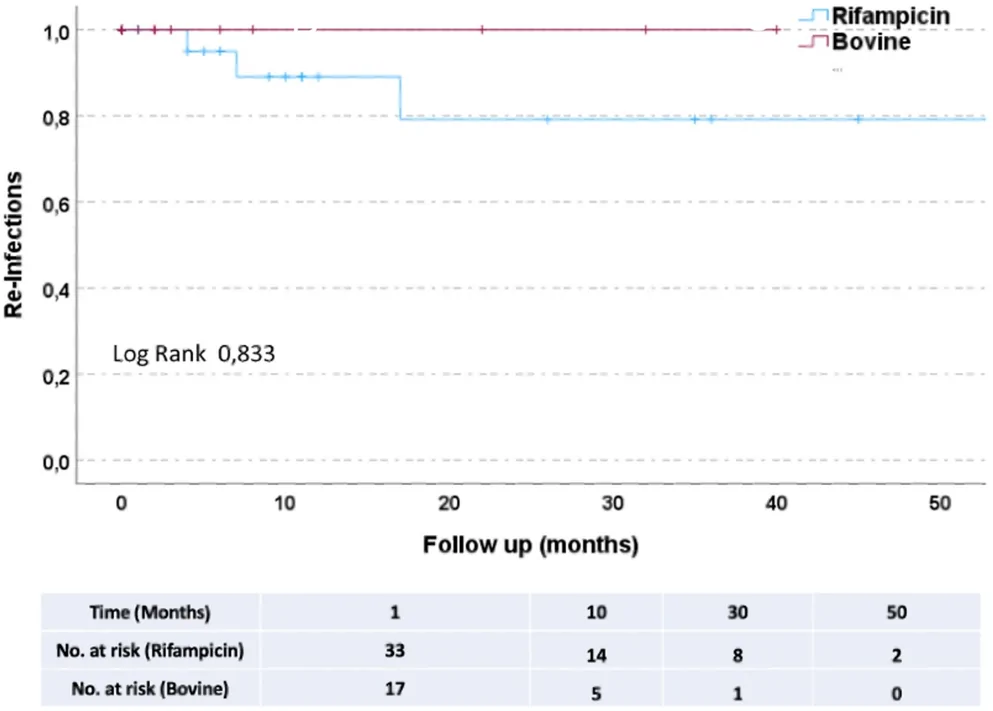
Kaplan-Meier curve of reinfection

During follow-up, treatment failure occurred in four patients, all of whom had undergone in-situ reconstruction with a rifampicin-soaked Dacron prosthesis. All treatment failures occurred in patients treated with rifampicin-soaked prostheses (4/41; 9.76%), whereas no treatment failures were observed in the bovine pericardial group (0/21; 0%) (*p* = 0.562) (Table 3). Kaplan–Meier analysis also revealed no significant differences between groups with regard to freedom from treatment failure (*p* = 0.833) (Fig. 4). The four treatment failures occurred after 17–84 months following reconstruction.

Microbiological analysis, based on preoperative blood cultures as well as intraoperative swabs and tissue specimens, identified Candida species as the most frequent pathogens overall (27.78%), followed by Escherichia coli (18.52%) and Enterococcus faecalis (14.81%). Pathogen detection was significantly more frequent in patients with secondary aorto-enteric fistula than in those without secondary aorto-enteric fistula (26/27 [96.30%] vs. 19/35 [54.28%], *p* < 0.001). Notably, Candida species were significantly more frequently detected in patients with secondary aorto-enteric fistula, being identified in 12 patients (*p* = 0.003). (Table 4)

**Table 4:** Microbiological findings in patients undergoing open surgical or endovascular treatment

Bacteria, *n* (%)	Total (*n* = 54)	open surgery (*n* = 31)	endovascular (*n* = 23)	*p*-value
E.coli	10 (18.52)	5 (16.13)	5 (21.74)	0.728
Klebsiella	5 (9.26)	2 (6.45)	3 (13.04)	0.64
S. aureus	7 (12.92)	3 (9.68)	4 (17.39)	0.443
S. capitis	1 (1.85)	0 (0)	1 (4.35)	0.426
S. haemophilus	1 (1.85)	1 (3.22)	0 (0)	1
E. faecalis	8 (14.81)	5 (16.13)	3 (13.04)	1
E. faecium	6 (11.11)	3 (9.68)	3 (13.04)	1
E. cloacae	2 (3.70)	2 (6.45)	0 (0)	0.502
Proteus mirabilis	2 (3.70)	0 (0)	2 (8.69)	0.177
Str. anginosus	5 (9.26)	3 (9.68)	2 (8.69)	1
Pseudomonas aeruginosa	4 (7.41)	4 (12.90)	0 (0)	0.127
Candida	15 (27.78)	10 (32.26)	5 (21.74)	0.541
Mycobacterium	0 (0)	0 (0)	0 (0)	0
Additional microbiological findings, n (%)		Patients with AEF (*n* = 27)	Patients without AEF (*n* = 35)	p-value
Pathogen detected, n (%)		26 (96.30)	19 (54.28)	< 0.001
No pathogen detected, n (%)		1 (3.70)	16 (45.71)	

In patients who had previously undergone open aortic repair, Candida species were detected in 32.26% of cases, whereas in patients with prior endovascular repair Candida accounted for 21.74%. Staphylococcus aureus was slightly more frequent in patients with previous endovascular procedures (17.39%) compared with those after open repair (9.68%). Enterococcus faecalis and Enterococcus faecium were identified in both groups without relevant differences.

Gram-negative organisms such as Escherichia coli and Klebsiella species were present in both cohorts without significant variation. Enterobacter cloacae was detected only in patients with previous open repair (6.45%). Pseudomonas aeruginosa occurred exclusively in the open repair cohort (12.90%). No Mycobacterium species were detected.

Overall, there were no statistically significant differences in the distribution of pathogens between patients with prior open versus endovascular aortic repair (Table 4).

Microbiological cultures remained negative in 8 of 62 patients (12.90%), indicating that no causative pathogen could be identified in these cases.

## Discussion

Aortic vascular graft and endograft infection (aortic VGEI) remains one of the most challenging complications after aortic repair, associated with high morbidity and mortality despite advances in surgical and endovascular techniques [1, 2] .

The main finding of this study is that treatment failures occurred exclusively in patients reconstructed with rifampicin-soaked Dacron prostheses, whereas no treatment failures were observed in patients treated with bovine pericardial grafts. The primary endpoint of this analysis was the occurrence of treatment failure after graft explantation and in-situ reconstruction. Secondary endpoints included major postoperative complications such as dialysis dependency, graft occlusion, amputation, and in-hospital mortality. In the present cohort, treatment failure occurred in 4 patients (9.76%) in the rifampicin group, whereas no treatment failures were observed in the bovine pericardial group.

Baseline characteristics did not differ significantly for most variables; however, several clinically relevant numerical imbalances were present between the groups. Given the small sample size and limited statistical power, the absence of statistical significance should not be interpreted as evidence of equivalence or comparability. The bovine cohort showed a higher proportion of patients with preoperative dialysis, whereas other relevant comorbidities such as advanced heart failure (NYHA III–IV) were not more frequent. These baseline imbalances may have influenced the observed outcomes and should be considered when interpreting comparisons between the two conduit groups.

Aortic cross-clamp time was significantly longer in patients treated with bovine grafts. The longer aortic clamping time observed in the bovine group may partly be explained by the fact that bovine pericardial grafts had to be individually prepared intraoperatively by the operating surgeon before implantation. However, differences in clamping levels between groups should be interpreted cautiously. During the study period, operative strategies evolved, and surgical decision-making regarding clamping technique was influenced by anatomical considerations and procedural planning. Consequently, differences in suprarenal or supravisceral clamping rates may reflect variations in operative strategy rather than intrinsic differences in disease severity. Operative urgency represents an important potential confounder in the treatment of aortic VGEI. In the present cohort, all patients underwent either urgent surgery within 24 h or immediate emergency surgery. This distinction is clinically relevant because haemodynamic instability, ongoing sepsis, or bleeding may restrict the time available for operative preparation and may influence conduit selection. An immediately available rifampicin-soaked prosthesis may be advantageous when rapid reconstruction is required, whereas preparation of a hand-sewn bovine pericardial conduit requires additional operative time. Accordingly, conduit-related outcomes should be interpreted in the context of operative urgency, and the corresponding subgroup results are considered exploratory because of the limited sample size.

Although no treatment failure were observed in the bovine pericardial group, the difference between treatment groups was not statistically significant. This finding should therefore be interpreted as a descriptive signal that may support further investigation rather than as evidence of superior resistance of biological graft material to recurrent infection. The observed difference may also have been influenced by differences in case mix, the non-randomized selection of conduit material, the shorter follow-up in the bovine group, and changes in institutional practice over time. Residual confounding and confounding by treatment era therefore cannot be excluded, and no causal relationship between graft material and treatment failure risk can be inferred from the present analysis [4, 9, 10]. The reduced treatment failure rate in the bovine group may be explained by the superior biocompatibility and lower bacterial adherence of biological material compared with synthetic prostheses. Similar outcomes have been reported in the literature, demonstrating excellent infection control and long-term patency of bovine pericardial grafts in infected aortic fields [9, 10]. In contrast, treatment failure rates of approximately 8–20% have been dscribed after prosthetic or rifampicin-soaked graft reconstructions, which may be related to limited antibiotic elution, biofilm formation, and the development of rifampicin resistance over time [8, 9, 15, 16]. The occurrence of treatment failure only in the rifampicin group is consistent with this previously reported signal; however, the low number of events and the absence of a statistically significant difference preclude firm conclusions.

Mortality remains substantial in patients undergoing surgery for aortic VGEI. In the present cohort, in-hospital mortality was 17.74%, with no significant difference between patients treated with bovine pericardial grafts (14.28%) and those receiving rifampicin-soaked prostheses (19.51%). These results appear slightly more favourable than previously reported perioperative mortality rates ranging between 20% and 40% for surgical treatment of aortic VGEI [1, 2, 16]. 

Most deaths occurred during the early postoperative period and were primarily related to septic complications or multiorgan failure associated with advanced graft infection. Mortality during longer-term follow-up was also frequently associated with persistent infection or severe systemic complications rather than with graft-related mechanical problems. These findings are concordant with larger series indicating that long-term survival is determined primarily by patient comorbidity, hemodynamic status, and infection severity at presentation rather than the type of graft used [16]. 

All procedures in this study were performed in a tertiary, high-volume vascular centre with established multidisciplinary expertise in the management of aortic VGEI. Previous reports have suggested that treatment in specialized centres with dedicated vascular surgery, infectious diseases, and intensive care support is associated with improved outcomes in this complex patient population. Our results support the concept that centralization of aortic VGEI care and high local expertise can achieve acceptable treatment failure and mortality rates despite the severity of disease [17]. 

Prolonged, targeted antimicrobial therapy is a cornerstone of aortic VGEI management. In our cohort, both groups were managed according to the same multidisciplinary antimicrobial principles, with intravenous treatment during the inpatient phase followed, in most cases, by prolonged oral therapy for approximately 8–12 weeks. Preoperative blood cultures were obtained before initiation of antimicrobial therapy. Treatment was adapted to microbiological findings, susceptibility testing, clinical response, laboratory parameters, and the adequacy of surgical source control, with antifungal therapy used when fungal pathogens were identified. Because both groups were treated according to the same institutional antimicrobial principles, major differences in the overall treatment strategy are unlikely to explain the observed differences in treatment failure. Current literature advocates an initial phase of intensive intravenous therapy followed by long-term, and in selected high-risk patients even lifelong, oral suppressive therapy after reconstruction for aortic VGEI [1]. Our practice is broadly consistent with these recommendations, and the favourable treatment failure profile of bovine pericardial grafts observed in our study supports their use within such standardized antimicrobial protocols.

Microbiological results showed Candida species as the most frequent pathogens, followed by Staphylococcus aureus and Escherichia coli (Table 4). This distribution is in line with recent studies highlighting the increasing role of fungal infections in aortic VGEI [4, 9]. Aortic VGEI complicated by secondary aorto-enteric fistula represents a distinct clinical entity with different microbiological characteristics, operative requirements, antimicrobial management, and prognosis. Accordingly, findings for patients with and without secondary aorto-enteric fistula were interpreted separately. Accurate diagnostic work-up is crucial, and while CT angiography remains the gold standard, PET-CT may provide additional sensitivity in cases with inconclusive findings [14, 18]. 

Secondary aorto-enteric fistula represents a particularly severe phenotype of aortic VGEI and may be associated with greater microbial burden, bleeding, sepsis, renal dysfunction, and operative complexity. Nevertheless, patients with AEF were intentionally retained in the overall cohort because they constituted a substantial proportion of the real-world aortic VGEI population treated at our center and because recurrent infection was the primary endpoint of this study. The central aim was to evaluate whether recurrent infection differed according to the implanted conduit rather than to exclude a common and clinically important manifestation of VGEI. Importantly, AEF was similarly distributed between the bovine pericardial and rifampicin-soaked Dacron groups (47.6% vs. 41.5%, *p* = 0.789), making a major imbalance in enteric involvement between conduit groups unlikely. Because AEF may nevertheless independently worsen postoperative outcomes, patients with and without AEF were additionally assessed in an exploratory subgroup analysis. Given the limited sample size, these subgroup findings should be interpreted descriptively and do not establish equivalence between the clinical phenotypes.

Overall, our findings suggest that bovine pericardial grafts may provide acceptable infection control without an apparent increase in perioperative risk. Given the retrospective design, limited sample size, unequal follow-up, and potential confounding by treatment era, these results should be regarded as descriptive and hypothesis-generating. When combined with surgery in a high-volume centre and standardized long-term antibiotic therapy, bovine pericardial in-situ reconstruction represents a reliable alternative to synthetic rifampicin-soaked prostheses for patients with aortic VGEI.

### Limitations

Our analysis was a retrospective, single-centre cohort study, which limits the generalizability of its conclusions. In addition, the small sample size, particularly in the bovine pericardial group, is a relevant limitation. The relatively short follow-up interval represents another risk of bias, as long-term durability and treatment failure rates may be underestimated. Furthermore, graft choice was not randomized but based on surgeon preference and patient condition, which may have introduced selection bias. Owing to the retrospective design, detailed information on individual antimicrobial agents, intravenous and oral treatment phases, and total treatment duration was not consistently available for standardized comparative analysis. Although antimicrobial treatment in both groups followed the same institutional multidisciplinary principles, residual differences in antimicrobial exposure cannot be excluded. Complete retrospective KDIGO staging was not feasible; renal outcomes were therefore reported descriptively according to preoperative dialysis dependency and postoperative dialysis requirement. Furthermore, the exploratory analyses according to operative urgency and AEF status were limited by small subgroup sizes and should be considered hypothesis-generating. Microbiological assessment included preoperative blood cultures as well as intraoperative swabs and tissue samples, which were processed using conventional culture techniques according to institutional standards. Nevertheless, reliance on culture-based methods may still underestimate culture-negative infections or atypical pathogens.

## Conclusions

These results suggest that bovine pericardial grafts represent a safe and durable alternative for in-situ reconstruction in patients with aortic graft infection. However, long-term durability remains uncertain, and larger multicentre studies with extended follow-up are necessary to confirm these promising findings.

## Data Availability

The data analyzed in this study are derived from hospital records and contain sensitive patient information. Therefore, the datasets generated and/or analyzed during the current study are not publicly available due to data protection regulations and patient confidentiality.
